# Cardiac Arrhythmia in a Patient with Sickle Cell Anemia and Falciparum Malaria Treated with Intravenous Artesunate

**DOI:** 10.1155/2019/1913685

**Published:** 2019-11-13

**Authors:** Abdulrahman Hummadi, Sultan Mubarki, Ayel yahay, Awad Mohammed Al-Qahtani

**Affiliations:** ^1^Internal Medicine and Endocrinology Consultant, Samtah General Hospital, Jizan, Saudi Arabia; ^2^King Fahad Central Hospital, Jizan, Saudi Arabia; ^3^Department of Medicine, Hematology Division, King Khalid University, Abha, Saudi Arabia; ^4^Department of Family and Community Medicine, College of Medicine, Najran University, Najran, Saudi Arabia

## Abstract

Treatment of severe malaria with artemisinin derivatives in patients with comorbid conditions such as sickle cell anemia must be considered with precaution. We report here a case of possibly undocumented ventricular arrhythmia in a sickle cell anemia patient diagnosed with *Plasmodium falciparum* malaria and treated with intravenous artesunate. The patient suffered from wide complex tachycardia after treatment with artesunate 170 mg (2.4 mg/kg) i.v. bolus, tachycardia was managed with amiodarone (150 mg i.v. for 10 minutes). Electrocardiographic abnormalities, including QT prolongation, are common in patients with sickle cell anemia. The mortality rate in sickle cell anemia patients due to cardiovascular and pulmonary complications remains high. The probability of precipitation of ventricular arrhythmias may increase in patients with sickle cell anemia, diagnosed with malaria and treated with artemisinin derivatives.

## 1. Introduction

Malaria remains one of the major public health issues in the modern world. Malaria is endemic mainly in the sub-Saharan Africa, Southeast Asia and Western Pacific regions. The WHO report shows that, in 2017, an estimated 219 million cases of malaria occurred worldwide, compared with 239 million cases in 2010 and 217 million cases in 2016. There were an estimated 435,000 malaria-related deaths in 2017 [1–3]. In 1948, Kingdom of Saudi Arabia (KSA) introduced the first extensive malaria control program, which led to sharp decline in the incidence of malaria. In 2017, the total number of indigenous confirmed malaria cases in Saudi Arabia was 177. The major *Plasmodium* species was *P. falciparum* (97%) followed by *P. vivax* (2%). There are members of four *Anopheles* species complexes in KSA including *An*. *gambiae*, *An. fluviatilis*, *An. culicifacies*, and *An. subpictus* complexes. The species of *An. arabiensis* from *An*. *gambiae* complex is the only member of the complex that extends outside of Africa into the southwestern and western areas of the Arabian Peninsula including in KSA and Yemen, where it is a major malaria vector. Other recorded vectors of malaria in KSA include *An. fluviatilis*, *An. stephensi*, and *An. azaniae* [4]. However, Saudi Arabia is facing the menace of malaria importation through millions of pilgrims coming from all over the world to perform Hajj every year and through emigrants who form the workforce of the country [5]. Successful malaria control depends greatly on treatment with efficacious antimalarial drugs. Artemisinin combination therapies (ACTs) are the first-line treatment for uncomplicated *Plasmodium falciparum* malaria recommended by the WHO. In adults, parenteral artesunate remains the treatment of choice for severe *P. falciparum* malaria [1]. However, these antimalarials, including quinidine, artemether, plus lumefantrine are notorious at producing cardiac dysrhythmias, QT interval prolongation on the electrocardiogram [6]. Halofantrine was considered an effective and safe treatment for multidrug resistant falciparum malaria until 1993, when the first case of drug-associated death was reported. Since then, numerous studies have confirmed cardiac arrythmias, possibly fatal, in both adults and children [7].

Chronic hemolytic anemia is the characteristic feature of sickle cell anemia. It is associated with high morbidity and mortality. Highest prevalence of sickle cell anemia in Saudi Arabia is in the Eastern Province, followed by the provinces of the southwestern region [8]. Electrocardiographic abnormalities, including QT prolongation and torsades de pointes, are commonly reported in patients with sickle cell anemia [9]. Sickle cell anemia can be a predisposing factor for precipitation of fatal cardiac arrhythmias in malaria patients treated with intravenous artesunate; here, we report a possibly undocumented ventricular tachycardia in a *P. falciparum* malaria patient with sickle cell anemia, treated with intravenous artesunate. A written informed consent of the patient was obtained to publish his data.

## 2. Case Report

A 19-year-old male with sickle cell anemia, being treated with folic acid, presented to the emergency department of Samtah General Hospital, Jizan, Saudi Arabia, with fever, nausea, headache, and yellowish discoloration of sclera. Patient was living in Jizan, an endemic area of malaria, which raised suspicion of malaria infection. Patient looked unwell, conscious, and alert. His vital signs were normal except the temperature which was high (38.5 degree C). Chest examination was normal and the cardiovascular system showed normal first and second heart sounds with no added sound. Fundus examination was normal. Other systemic reviews were unremarkable. Patient was admitted in the medical ward, and baseline laboratory investigations revealed the following remarkable laboratory values: white blood cells 12 × 10^9^/L (normal range: 4.5 to 11.0 × 10^9^/L); hemoglobin 7.3 g/dL (normal range: 13.8 to 17.2 g/dL); mean corpuscular volume 72 fL/red cell (normal range: 80–96 fL/red cell); platelet count 62 × 10^3^/*μ*L (normal range: 150–400 × 10^3^/*μ*L); reticulocyte 2.6% (normal range: 0.5% to 2.5%); aspartate transaminase 89 U/L (normal range: 10 to 40 U/L); alanine transaminase 78 U/L (normal range: 7 to 56 U/L); total bilirubin 600 *μ*mol/L (normal range: 1.71 to 20.5 *μ*mol/L); direct bilirubin 200 *μ*mol/L (normal range: less than 5.1 *μ*mol/L); and sodium 149 mmol/L (normal range: 136–145 mmol/L), whereas the following laboratory values were found to be in normal ranges: alkaline phosphatase 120 IU/L (normal range: 44 to 147 IU/L); random blood glucose 7.2 mmol/L (normal range: 4.4–8.9 mmol/L); potassium 4.9 mmol/L (normal range: 3.6–5.2 mmol/L); magnesium 0.9 mmol/L (normal range: 0.6–1.1 mmol/L); calcium 2.3 mmol/L (normal range: 2.2–2.7 mmol/L); phosphate 1.4 mmol/L (normal range: 1.12–1.45 mmol/L); creatinine 78 mmol/L (normal range: 60–110 mmol/L); and urea 3.7 mmol/L (normal range: 2.5–7.1 mmol/L).

**Figure 1:**
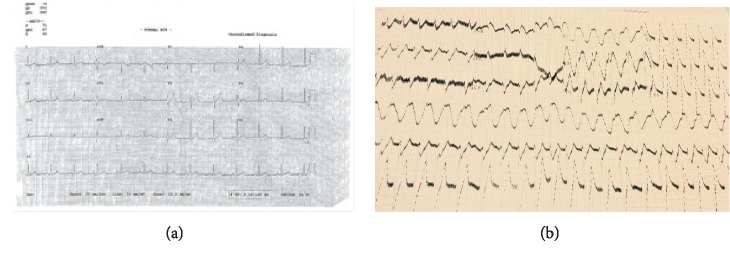
(a) Baseline ECG of the patient; (b) ECG showing ventricular tachycardia after artesunate administration.

ECG initially showed normal sinus rhythm, and chest X-ray was normal. The patient had no previous history of cardiac arrhythmias, and his baseline ECG was normal (Figure 1(a)). Peripheral blood smear revealed *P. falciparum* (ring form) with parasitemia index 10%. His body weight was 74 kg and height 166 cm.

Patient was diagnosed with severe *P. falciparum* malaria based on clinical presentation and laboratory investigations and started on intravenous artesunate 170 mg (2.4 mg/kg) bolus slowly over 2 minutes at 0 hour, 12 hours, and 24 hours, and every 24 hours thereafter for 5 days. The powder for injection was reconstituted with 5% sodium bicarbonate and diluted in an equal volume of normal saline. He was started on paracetamol 500 mg tablet orally as needed every 6 hours. Patient was clinically improving and temperature subsided. After 4 hours of the third dose of artesunate, the patient became sick, faint, and suddenly arrested. Resuscitation was done for 32 minutes, ECG was done and showed wide complex ventricular tachycardia, and QTc (Bazett) was 516 msec (Figure 1(b)). Electrolytes were in the normal limits: sodium 136 mmol/L; potassium 4.3 mmol/L; magnesium 0.8 mmol/L; and calcium 2.1 mmol/L. Tachycardia was managed by administration of amiodarone (150 mg i.v. for 10 minutes). The patient revived and kept in ICU for further observation. There was no worsening of hemolytic anemia during and after the treatment with artesunate. The treatment plan was not changed; the next dose of artesunate was after 24 hours of the third dose (last dose given before event happened). The patient clinically improved, fever subsided, and the temperature decreased with paracetamol to 37.4°C, and he was walking and conversing normally. The treatment was completed by oral artesunate 100 mg + sulfadoxine 500 mg/pyrimethamine 25 mg tablets (3-day course).

## 3. Discussion

Malaria is the one of the major health problems facing the world. The most common species worldwide is *P. falciparum*, which represents more than 90% of locally acquired malaria in the Arabian Peninsula. In Saudi Arabia, the highest incidence of *P. falciparum* malaria has been reported in the south-west provinces of Aseer and Jizan [5]. It is one of the countries that is facing the challenge of malaria elimination.

Malarial parasite is able to develop resistance against antimalarial drugs. Hence, WHO recommends the use of combinations of two drugs, having different mechanisms of action, as a strategy to overcome antimalarial resistance. Artemisinin is one of the vital components of antimalarial combination therapy, known as artemisinin-based combination (ACTs). It is now considered as first-line treatment for complicated and uncomplicated malaria in Saudi Arabia. Our case diagnosed as complicated *P. falciparum* malaria in known patient of sickle cell anemia and started on intravenous artesunate, as per the national policy of malaria case management in the Kingdom of Saudi Arabia [10]. It is relatively a safe drug and not known to cause QT prolongation; however, QT prolongation has been reported with other artemisinin derivatives such as artemether [6]. In a previous study conducted at Chittagong Medical College Hospital, a large 1,000-bed teaching hospital in Chittagong, Bangladesh, it has been reported that the mean QTc interval was unaffected by bolus intravenous artesunate (2.4 mg/kg) in *P. falciparum* malaria patients. However, in only two patients (*n* = 21), the QTc interval exceeded 0.5 seconds, but in both cases, an alternative explanation was plausible [11]. In another report, the QTc lengthening episode and transient bradycardia possibly due to artesunate therapy have been reported in one and two patients, respectively [12]. Alteration in calcium metabolism can occur in malaria patients. Hypocalcaemia in malaria can lead to prolonged QTc interval, which in turn can be a risk factor for quinine cardiotoxicity and sudden death. Hence, any drug which can cause QTc interval prolongation should be used with caution in such patients [13]. One report suggests that the mean QTc interval was unaffected by artesunate [11]. Artesunate is an investigational drug and not yet approved in the US and only available through an Investigational New Drug (IND) protocol [1]; however, it is not so in Asia, Sahara, and sub-Sahara where malaria is a scourge. There are eligibility criteria under the IND protocol to use it in patients with malaria. Scientific evidence reveals no relationship between artesunate and arrhythmia; however, we present a case of cardiac arrhythmia, possibly related to artesunate therapy. In our case, after 4 hours of the third dose, patient became sick, faint and suddenly arrested. Resuscitation was done for 32 minutes; ECG showed wide complex ventricular tachycardia. Artesunate terminal elimination half-life (T1/2) is 0.25 (0.1–1.8) hours, and for its active metabolite dihydroartemisinin T1/2 is 1.31 (0.8–2.8) hours [14]. It has been previously reported in patients with uncomplicated falciparum malaria that elimination of artesunate and its active metabolite dihydroartemisinin is essentially complete 6 hours after an i.v. injection of artesunate [15]. There are no known interactions between artesunate and other drugs. [3] Moreover, there were no other medications known to induce these cardiac effects during treatment. Thus, the causality of the cardiac event could probably be attributed to artesunate. However, the other predisposing factors in this case, such as sickle cell anemia and severe malaria itself, have been known to cause arrhythmias. This occurrence of arrhythmia may not only be relevant for malaria treatment but also for treatment of other diseases with artesunate, as it is now well established that artesunate and *Artemisia annua* are also active against other diseases, such as, cancer, viral infections, schistosomiasis, and trypanosomiasis. Artesunate has been effectively and safely used as an add-on therapy to the guideline-based oncological therapy in patients with metastatic breast cancer [16]. In a recent study, dried leaf *Artemisia annua* demonstrated efficacy against non-small cell lung cancer cells [17]. Thus, the occurrence of cardiac events in these groups is plausible and should be kept in mind prior to utilization of artesunate.

## 4. Conclusion

Due to presence of other predisposing factors known to cause arrhythmias, it is presumed that artesunate induced the ventricular arrhythmia. Artesunate is a relatively new antimalarial drug from artemisinin derivatives. It is considered as first-line treatment in patients with malaria in many endemic areas. However, the possibility of cardiovascular effects of artesunate should always be kept in mind prior to its administration, especially in patients with predisposing factors for causing arrhythmias, such as sickle cell anemia and in malaria-endemic regions.
